# Fluorescence-Guided Surgery of Liver Metastasis in Orthotopic Nude-Mouse Models

**DOI:** 10.1371/journal.pone.0138752

**Published:** 2015-10-01

**Authors:** Takashi Murakami, Yukihiko Hiroshima, Yong Zhang, Takashi Chishima, Kuniya Tanaka, Michael Bouvet, Itaru Endo, Robert M. Hoffman

**Affiliations:** 1 AntiCancer, Inc., San Diego, California, United States of America; 2 Department of Surgery, University of California San Diego, San Diego, California, United States of America; 3 Department of Gastroenterological Surgery, Graduate School of Medicine, Yokohama City University, Yokohama, Japan; Stanford University, UNITED STATES

## Abstract

We report here the development of fluorescence-guided surgery of liver metastasis. HT29 human colon cancer cells expressing green fluorescent protein (GFP) were initially injected in the spleen of nude mice. Three weeks later, established liver metastases were harvested and implanted on the left lobe of the liver in other nude mice in order to make an orthotopic liver metastasis model. Fourteen mice with a single liver metastasis were randomized into bright-light surgery (BLS) or fluorescence-guided surgery (FGS) groups. Seven mice were treated with BLS, seven were treated with FGS. Three weeks after implantation, the left lobe of the liver with a single metastasis was exposed through a median abdominal incision. BLS was performed under white light. FGS was performed using a hand-held portable fluorescence imaging system (Dino-Lite). Post-surgical residual tumor fluorescence was visualized with the OV100 Small Animal Imaging System. Residual tumor fluorescence after BLS was clearly visualized at high magnification with the OV100. In contrast, residual tumor fluorescence after FGS was not detected even at high magnification with the OV100. These results demonstrate the feasibility of FGS for liver metastasis.

## Introduction

The ability of the surgeon to accurately visualize tumor margins is essential at the time of surgery and is of particular importance for resection of metastatic disease, especially in the liver [1].

A variety of labeling compounds have been used for fluorescence-guided surgery in the clinic. Sentinel lymph nodes in breast cancer patients were labeled by a near-infrared (NIR) fluorescing dye indocyanine [2]. However, indocyanine does not specifically label tumor cells. The metabolite 5-aminolevulinic acid, a precursor of hemoglobin, labels porphyrins in malignant glioma for FGS [3], which significantly improved outcome.

Folate was coupled to fluorescein isothiocyanate (FITC) for targeting folate receptor–α (FR-α) in ovarian cancers. Under fluorescence-guided surgery, tumor deposits less than 1 mm in size could be visualized and resected [4].

Monoclonal antibodies directed against cancer antigen 19–9 (CA19-9) or carcino-embryonic antigen (CEA) were conjugated to a green fluorophore and delivered intravenously into nude mice with orthotopic human pancreatic or colon tumors [5]. The tumors become fluorescent and were resected under fluorescence-guidance improved outcome [5–10].

Kishimoto *et al*. [11] labeled tumors with green fluorescence protein (GFP) using a telomerase-dependent adenovirus (OBP-401) that expresses the *gfp* gene only in cancer cells, which, in contrast to normal cells, express the telomerase enzyme. The labeled tumors could also be resected under fluorescence guidance. Tumors that recurred after fluorescence-guided surgery maintained GFP expression [12]. Since the cancer cells stably express GFP, detection of cancer recurrence and metastasis is possible with OBP-401 GFP labeling, and is not possible with nongenetic probes.

The present report demonstrates the feasibility of FGS of liver metastasis labeled with GFP in orthotopic mouse models.

## Materials and Methods

### Ethics Statement

All animal studies were conducted with an AntiCancer Institutional Animal Care and Use Committee (IACUC)-protocol specifically approved for this study and in accordance with the principals and procedures outlined in the National Institute of Health Guide for the Care and Use of Animals under Assurance Number A3873-1. In order to minimize any suffering of the animals, anesthesia and analgesics were used for all surgical experiments. Animals were anesthetized by intramuscular injection of a 0.02 ml solution of 20 mg/kg ketamine, 15.2 mg/kg xylazine, and 0.48 mg/kg acepromazine maleate. The response of animals during surgery was monitored to ensure adequate depth of anesthesia. Ibuprofen (7.5 mg/kg orally in drinking water every 24 hours for 7 days post-surgery) was used in order to provide analgesia post-operatively in the surgically-treated animals. The animals were observed on a daily basis and humanely sacrificed by CO_2_ inhalation when they met the following humane endpoint criteria: prostration, skin lesions, significant body weight loss, difficulty breathing, epistaxis, rotational motion and body temperature drop. The use of animals was necessary to develop fluorescence-guided surgery of liver metastasis. Animals were housed with no more than 5 per cage. Animals were housed in a barrier facility on a high efficiency particulate arrestance (HEPA)-filtered rack under standard conditions of 12-hour light/dark cycles. The animals were fed an autoclaved laboratory rodent diet (S1 ARRIVE Checklist).

### Cell Line

The human colon cancer cell line HT-29 [10], obtained from the American Type Culture Collection (Rockville, MD) was maintained in DMEM (Irvine Scientific, Irvine, CA) supplemented with heat-inactivated 10% fetal bovine serum (FBS) (Gemini Biologic Products, Calabasas, CA), 2 mM glutamine, 100 units/ml penicillin, 100 μg/ml streptomycin, and 0.25 μg/ml amphotericin B (Life Technologies, Inc., Grand Island, NY). The cells were incubated at 37°C in 5% CO_2_.

### Mice

Athymic *nu/nu* nude mice (AntiCancer Inc., San Diego, CA), 4–6 weeks old, were used in this study. Mice were kept in a barrier facility under HEPA filtration (as noted above). Mice were fed with an autoclaved laboratory rodent diet. All mouse surgical procedures and imaging were performed with the animals anesthetized by subcutaneous injection of the ketamine mixture described above. All animal studies were conducted with an AntiCancer Institutional Animal Care and Use Committee (IACUC)-protocol specifically approved for this study and in accordance with the principals and procedures outlined in the National Institute of Health Guide for the Care and Use of Animals under Assurance Number A3873-1.

### Establishment of GFP-labeled HT29

For GFP gene transfection, 25% confluent HT-29 cells were incubated with a 1:1 mixture of GFP retroviral supernatants of PT67 packaging cells and RPMI 1640 cell culture medium (GIBCO Life Technologies, New. York, NY) containing 10% FBS (Gemini Biological Products) for 72 h. Fresh medium was replenished at this time. Cells were harvested by trypsin-EDTA 72 h after transduction and subcultured at a ratio of 1:15 into medium, which contained 200 μg/ml G418 to select for high GFP expression, since the GFP retrovirus also contained the neomycin-resistance gene. The level of G418 was increased to 400 μg/ml stepwise. Clones stably expressing GFP were isolated with cloning cylinders (Bel-Art Products, Pequannock, NJ) with the use of trypsin-EDTA. The high-GFP-expressing cells were then amplified and transferred by conventional culture methods. High GFP-expression clones were then isolated in the absence of G418 for 10 passages to select for stable expression of GFP [13–15].

### Initial Establishment of Liver Metastasis

**Fig 1:**
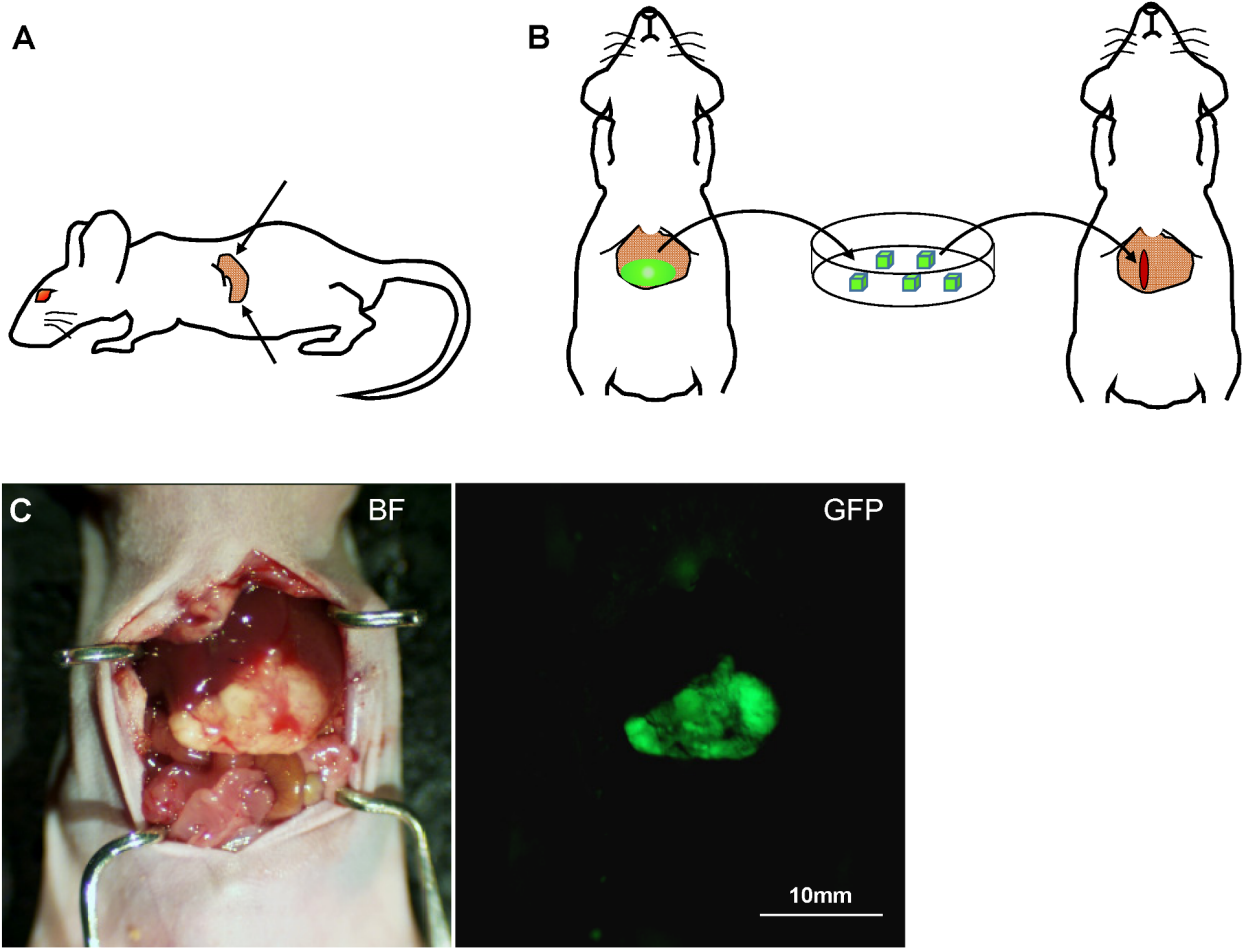
HT29-GFP colon cancer experimental liver metastasis. **(A)** HT-29-GFP cells (5×10^5^ in 50 μl with 50% Matrigel) were injected into the superior pole and inferior pole of the spleen (arrows), respectively. **(B)** Three weeks after injection, liver metastasis was confirmed by laparotomy, which was resected and cut into 3-mm^3^ blocks. Each tumor fragment was implanted by surgical orthotopic implantation (SOI) in the left lobe of the liver on other nude mice. **(C)** Representative images of liver metastasis established after spleen injection. The large tumor in the liver strongly expressed GFP. (GFP, green fluorescent protein; BF, bright field)

HT-29-GFP cells were harvested by trypsinization and washed twice with serum-free medium. Cells (5×10^5^ in 50 μl serum-free medium with 50% Matrigel) were injected into the superior and inferior pole of the spleen in mice (Fig 1A). Three week after injection, liver metastasis was confirmed by laparotomy (Fig 1C).

### Surgical orthotopic Implantation (SOI) of Liver Metastasis

Liver metastases were resected and cut into 8 mm^3^ blocks. A small 6- to 8-mm midline incision was made in other nude mice. The left lobe of the liver was exposed through this incision, and a single tumor fragment (3-mm^3^) was orthotopically implanted to the left lobe of the liver (Fig 1B). The left lobe of the liver was returned to the abdominal cavity, and the incision was closed in one layer using 6–0 nylon surgical sutures (Ethilon; Ethicon Inc., NJ, USA).

### Bright-Light and Fluorescence-Guided Surgery of Liver Metastases

**Fig 2:**
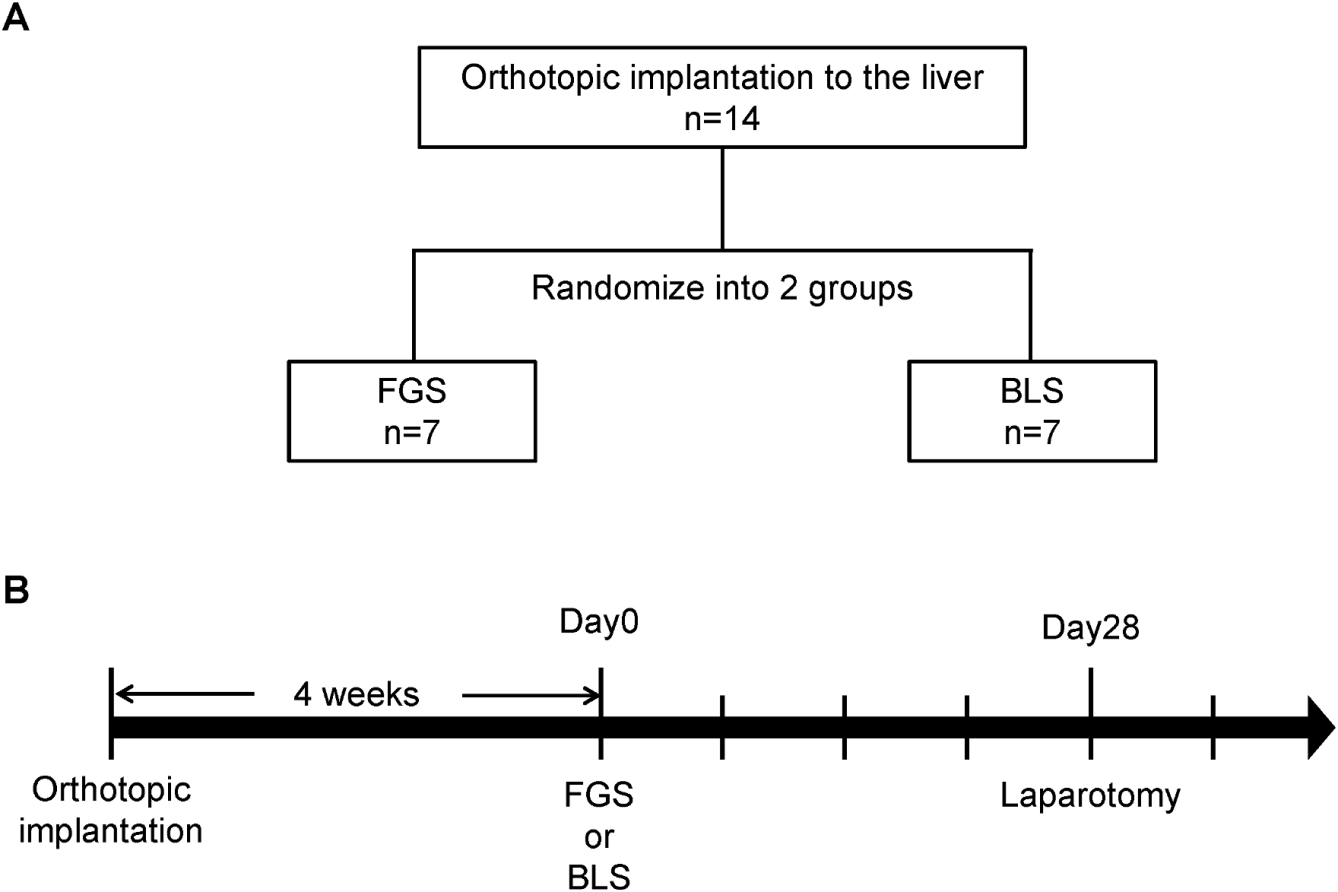
Schematic diagram of the experimental design. **(A)** Fourteen mice were randomized into 2 groups; FGS: n = 7, BLS: n = 7. **(B)** Timeline from orthotopic implantation. Four weeks after implantation, all mice were treated with FGS or BLS. Twenty-eight days after the surgery, all mice underwent laparotomy to detect GFP for evaluation of recurrence. After the laparotomy, follow-up examination for survival was continued. (FGS, fluorescence-guided surgery; BLS, bright-light surgery).

Four weeks after SOI of HT-29-GFP to the liver, the liver metastasis was exposed and imaged preoperatively with the OV100 Small Animal Imaging System (Olympus, Tokyo, Japan) [16] at a magnification of 0.14x. Fourteen mice underwent surgery: Fluorescence-guided surgery (FGS) in 7 mice and bright-light surgery (BLS) in 7 mice (Fig 2A and 2B). FGS was performed using the Dino-Lite imaging system (AM4113TGFBW Dino-Lite Premier; AnMo Electronics Corporation, New Taiwan). The surgical resection bed was imaged with the Olympus OV100 at a magnification of 0.14x or 0.56x to detect microscopic residual cancer. Residual tumor area was analyzed with ImageJ v1.49f (National Institutes of Health). The incision was closed in one layer using 6–0 nylon surgical sutures.

### Imaging of Tumor Progression

To evaluate tumor fluorescence around the resected site in the liver, laparotomy was performed in all mice at the 28^th^ post-operative day (Fig 2B). Tumor fluorescence was detected with the OV100 and analyzed with ImageJ.

### Tumor histology

Resected fresh tumor samples from either BLS or FGS were fixed in 10% formalin, and then embedded in paraffin. The tissue sections were deparaffinized in xylene and rehydrated in an ethanol series. Hematoxylin and eosin (H & E) staining was performed according to standard protocols. The margin between the tumor and the liver tissue was evaluated with a BHS System microscope (Olympus).

### Statistical Analysis

SPSS statistics version 21.0 was used for all statistical analyses (IBM, New York City, NY, USA). Residual tumor area is expressed as mean ± SD. Significant differences for continuous variables were determined using the Mann-Whitney U test. A probability value of *P* ≤ 0.05 was considered statistically significant.

## Results and Discussion

### FGS significantly reduces residual liver metastasis

**Fig 3:**
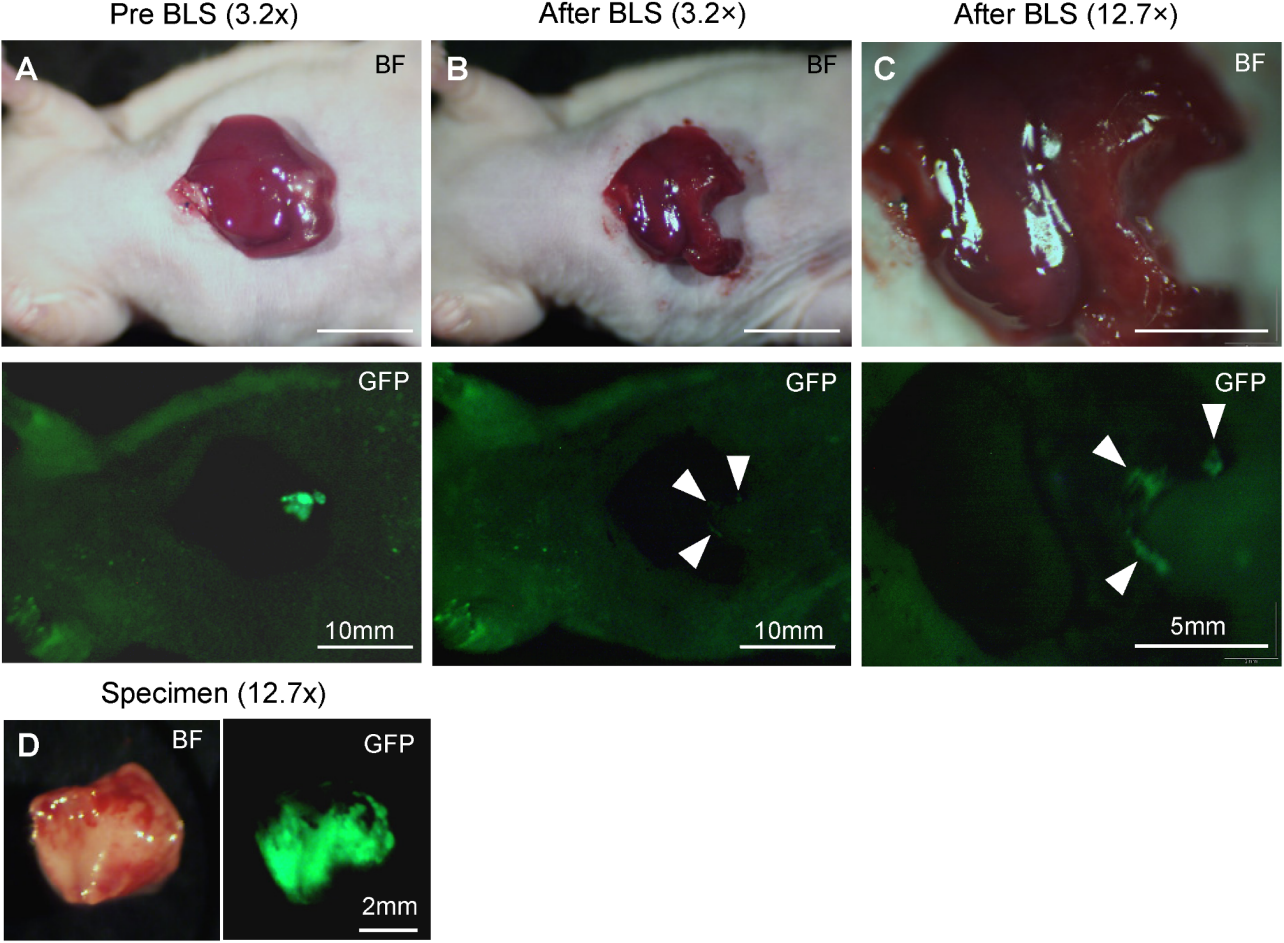
Pre-operative and post-operative images from the orthotopic liver metastasis model treated with BLS. **(A)—(C)** Upper panels show bright field images, and lower panels are images of tumor fluorescence obtained with the OV100. At low magnification, residual tumor fluorescence was marginally detected. **(B)** However, at high magnification, residual tumor fluorescence was clearly visualized (arrows) **(C)**. Arrowheads show residual tumor fluorescence in **B** and **C**. **(D)** Resected specimen. Magnifications are indicated above the columns.

**Fig 4:**
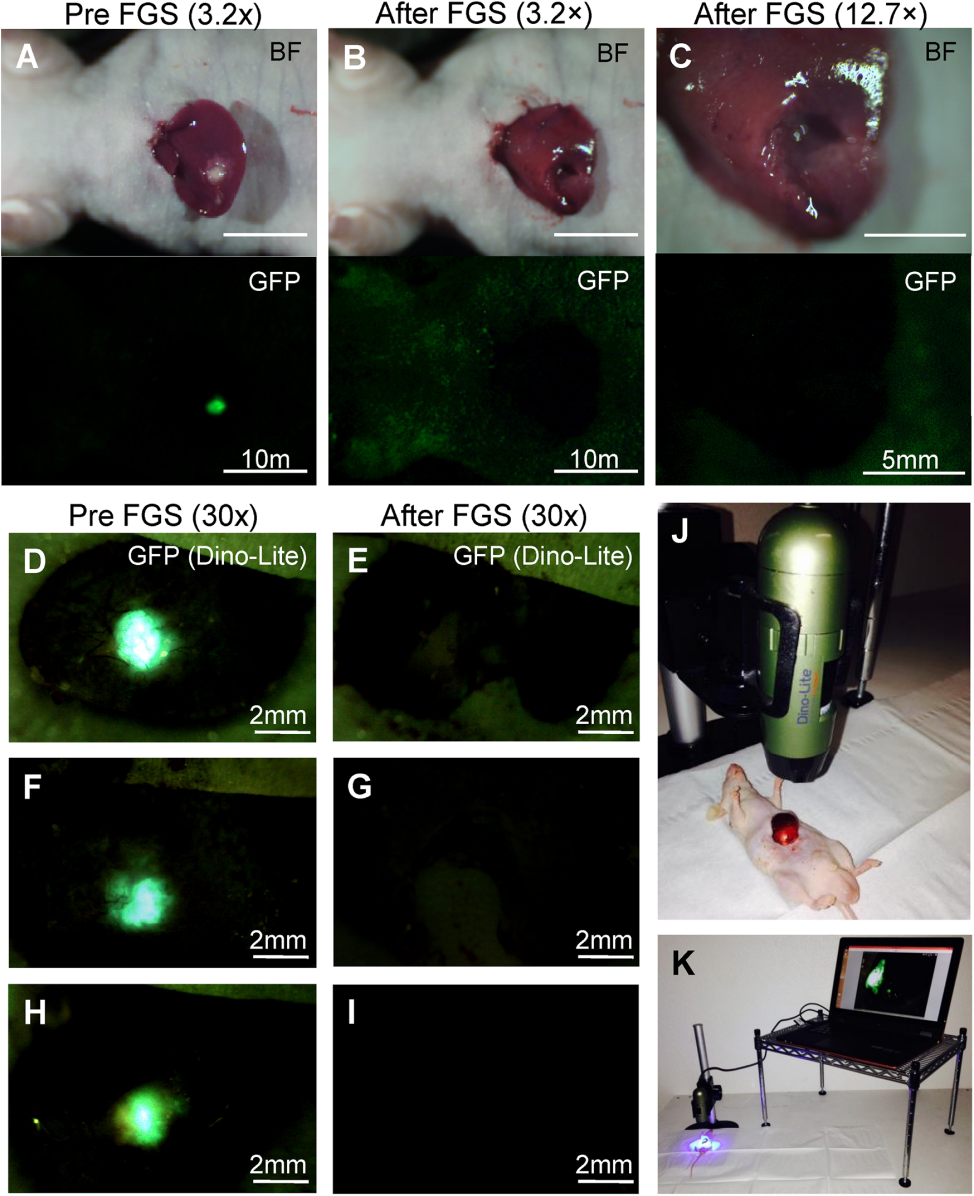
Pre-operative and post-operative images from the orthotopic liver metastasis model treated with FGS. **(A)—(C)** Upper panels show bright field images, and lower panels are images of tumor fluorescence obtained with the OV100. Residual tumor fluorescence could not be detected even at high magnification **(C). (D,F,H)** Pre-FGS tumor fluorescence was clearly visualized with the Dino-Lite imaging system. **(E,G,I)** Dino-Lite imaging showed no evidence of tumor after FGS. **(J-K)** Dino-Lite settings. **(J)** After exposing the left lobe of the liver, the mouse was put under the Dino-Lite. **(K)** Connection between the Dino-Lite and computer. Tumor fluorescence was imaged on the monitor during FGS. Magnifications are indicated above the columns.

**Fig 5:**
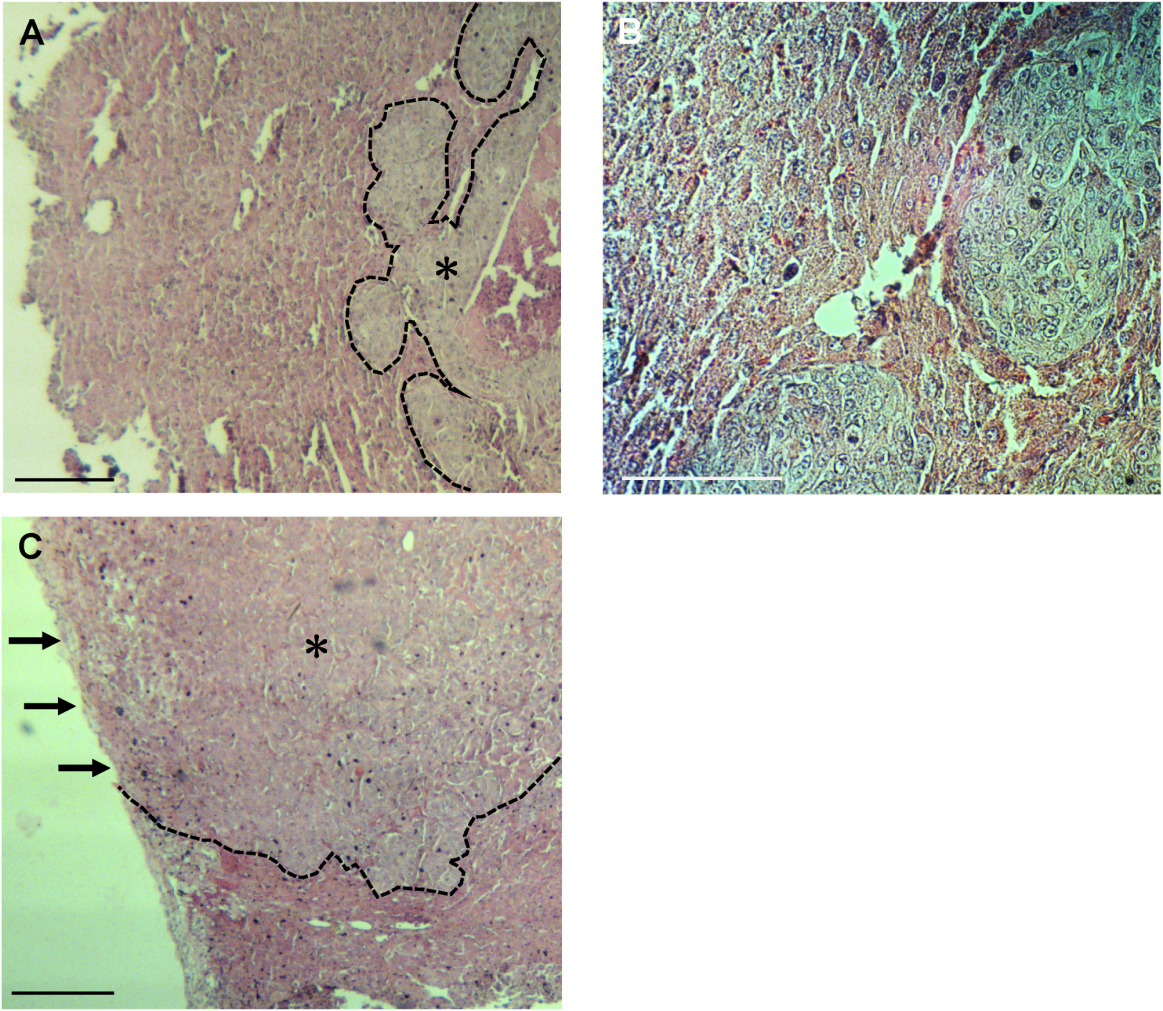
Histological tumor margin of resected specimen. **(A)-(C)** H&E staining of resected specimen. **(A)** In the mouse treated with FGS, viable cancer tissue (marked by an asterisk) is surrounded by normal liver tissues. **(B)** High magnification of **(A)**. **(C)** In the BLS-treated mouse, viable cancer cells are visible along the resection line. Arrows show residual cancer tissue. Dashed lines separate viable cancer and normal liver tissue. Scale bars: 200 μm.

**Fig 6:**
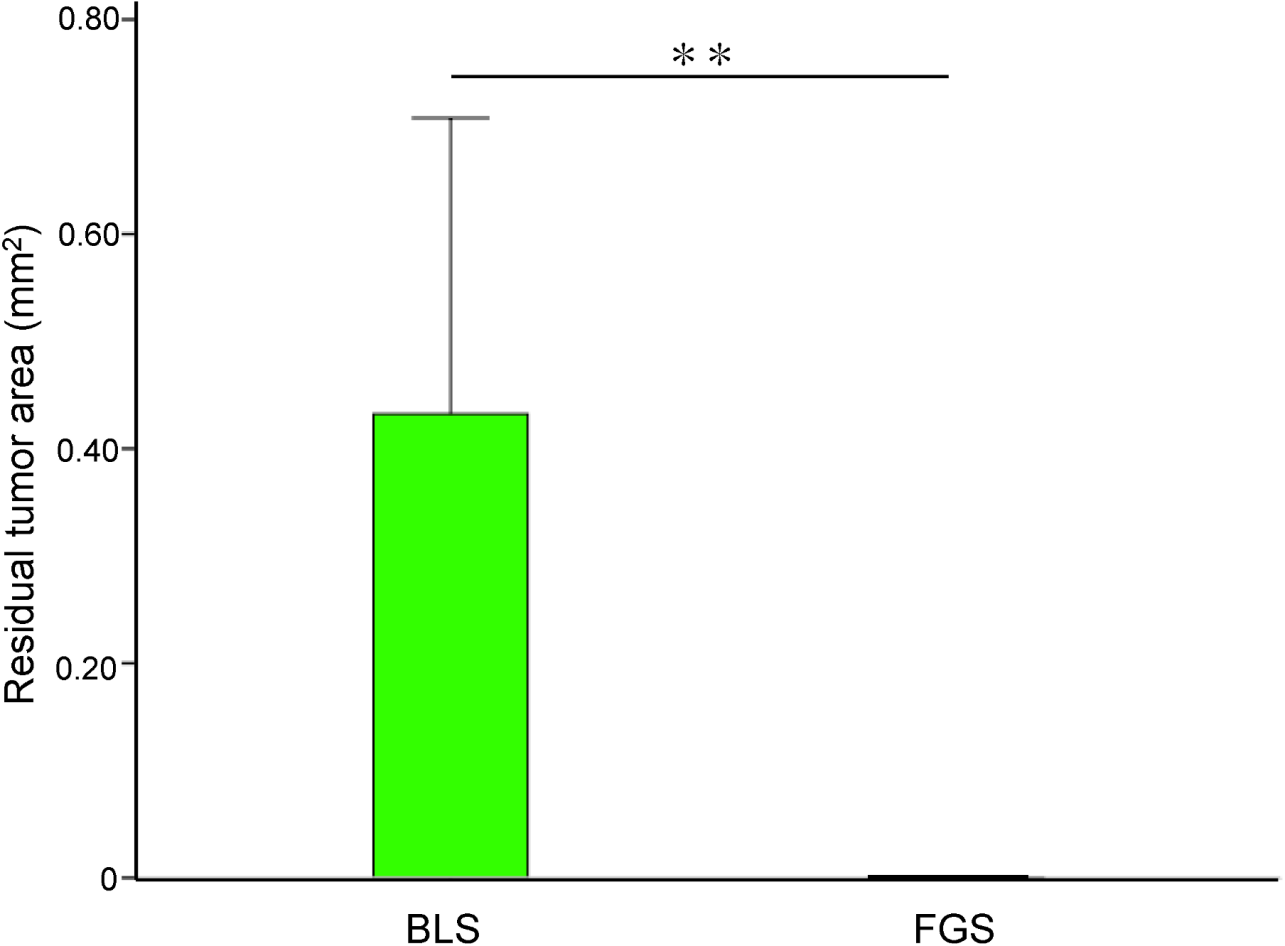
Bar graph of residual tumor area after surgery. No residual tumor was detected in the FGS group. Residual tumor area after BLS was relatively large. Error bar shows SD. **P<0.01.

Single liver metastases were clearly detected preoperatively with the OV100 (Figs 3A and 4A). In the BLS group, residual tumor fluorescence was marginally detected at a magnification of 0.14×. However, at a magnification of 0.56×, residual cancer was clearly visualized (Fig 3B and 3C). By contrast, residual tumor fluorescence could not be detected even at a magnification of 0.56× in the FGS group (Fig 4B and 4C). Tumor fluorescence was also clearly visualized before FGS with the Dino-Lite at a magnification of 30× (Fig 4D). Dino-Lite imaging showed no evidence of residual cancer after FGS (Fig 4E). All resected specimens exhibited strong GFP expression (Fig 3D). Histologically, viable cancer was detected on the resection line in the BLS group, but not in the FGS group which had clear margins (Fig 5), which is consistent with the fluorescence data. Residual tumor area after BLS was significantly larger than after FGS (0.34 ± 0.28 mm^2^ and 0 mm^2^, respectively; *P* = 0.003; Fig 6).

### FGS significantly decreases recurrent tumor area

**Fig 7:**
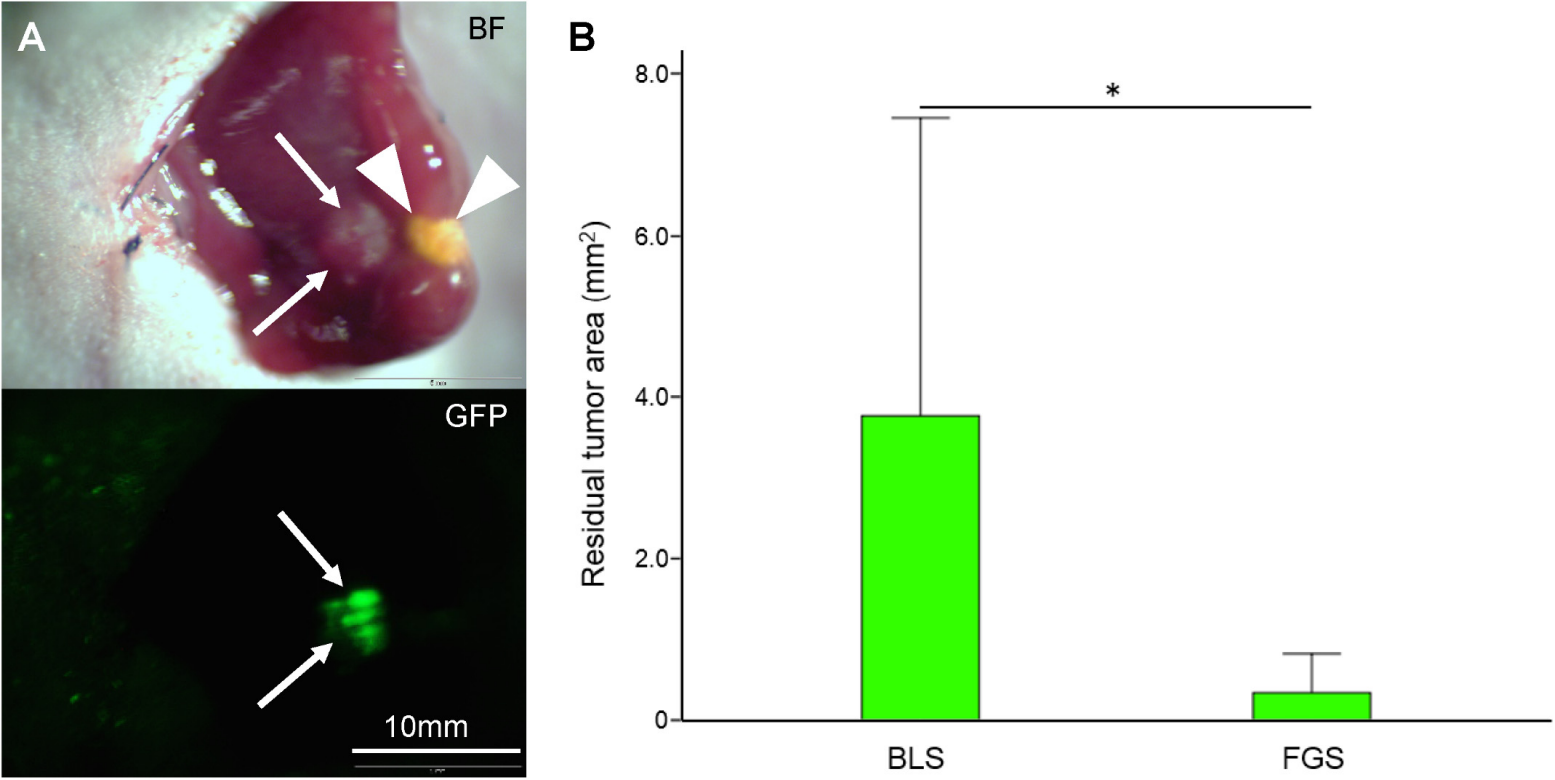
Evaluation of tumor fluorescence at day 28 after surgery. **(A)** Upper panel shows the bright field image, and lower panel shows the GFP tumor fluorescence image obtained with the OV100 at a magnification of 0.56. Laparotomy was performed at the 28^th^ postoperative day. Bright field image shows tumor in the resection site in the liver (arrows). Strong GFP fluorescence from the tumor is seen in the lower panel. Arrows show recurrent tumor in the resection site. Arrowheads show operative scar on the liver. **(B)** The GFP tumor fluorescence area was significantly larger in the BLS group compared to the FGS group, where only autofluorescence was detected. Error bars show SD. *P<0.05.

Laparotomy was performed at 28 postoperative day. Recurrent tumor fluorescence was visualized next to the resected site in the liver in the BLS group, but not in the FGS group (Fig 7). In the FGS group, only autofluorescence was detected.

The FGS procedure for liver metastasis described in the present report had short- and long-term advantages over BLS in that most if not all of the metastasis was resected by FGS compared to BLS (Figs 3–6), and this advantage was maintained even at 28 days post-surgery, where recurrence was greatly reduced by FGS compared to BLS (Fig 7). Future studies will devise curative strategies for FGS of liver metastasis.

## Conclusions

The results of present study suggest that FGS has clinical potential of reducing residual cancer in patients who have liver metastasis resected. Clinical application strategies could include labeling the liver metastasis with a tumor-specific monoclonal antibody such as anti-carcinoembryonic antigen (anti-CEA) conjugated to a fluorophore [5–10] or labeled by a telomerase-dependent cancer-specific adenovirus that contains the GFP gene, OBP-401 [11, 12, 17–19]. The labeled-antibody strategy is probably feasible in the near future. The viral-labeling strategy will require more safety studies. However, the parent virus of OBP-401, OBP-301, has proven safe in a clinical trial [20]. However, OBP-401 will also have safety tested in a clinical trial.

## Supporting Information

S1 ARRIVE Checklist (PDF)
